# Up-regulation of non-photochemical quenching improves water use efficiency and reduces whole-plant water consumption under drought in *Nicotiana tabacum*

**DOI:** 10.1093/jxb/erae113

**Published:** 2024-03-12

**Authors:** Benjamin Turc, Seema Sahay, Jared Haupt, Talles de Oliveira Santos, Geng Bai, Katarzyna Glowacka

**Affiliations:** Department of Biochemistry and Center for Plant Science Innovation, University of Nebraska-Lincoln, Lincoln, NE, USA; Department of Biochemistry and Center for Plant Science Innovation, University of Nebraska-Lincoln, Lincoln, NE, USA; Department of Biochemistry and Center for Plant Science Innovation, University of Nebraska-Lincoln, Lincoln, NE, USA; Department of Biochemistry and Center for Plant Science Innovation, University of Nebraska-Lincoln, Lincoln, NE, USA; Laboratory of Genetics and Plant Breeding, Universidade Estadual do Norte Fluminense – Darcy Ribeiro, Campos dos Goytacazes, RJ, Brazil; Department of Biological Systems Engineering, University of Nebraska-Lincoln, Lincoln, NE, USA; Department of Biochemistry and Center for Plant Science Innovation, University of Nebraska-Lincoln, Lincoln, NE, USA; Institute of Plant Genetics, Polish Academy of Sciences, 60-479 Poznań, Poland; University of Essex, UK

**Keywords:** Crop improvement, drought stress, *Nicotiana tabacum*, non-photochemical quenching (NPQ), red light stomatal regulation, tobacco, water use efficiency (WUE)

## Abstract

Water supply limitations will likely impose increasing restrictions on future crop production, underlining a need for crops that use less water per mass of yield. Water use efficiency (WUE) therefore becomes a key consideration in developing resilient and productive crops. In this study, we hypothesized that it is possible to improve WUE under drought conditions via modulation of chloroplast signals for stomatal opening by up-regulation of non-photochemical quenching (NPQ). *Nicotiana tabacum* plants with strong overexpression of the *PsbS* gene encoding PHOTOSYSTEM II SUBUNIT S, a key protein in NPQ, were grown under differing levels of drought. The *PsbS*-overexpressing lines lost 11% less water per unit CO_2_ fixed under drought and this did not have a significant effect on plant size. Depending on growth conditions, the *PsbS*-overexpressing lines consumed from 4–30% less water at the whole-plant level than the corresponding wild type. Leaf water and chlorophyll contents showed a positive relation with the level of NPQ. This study therefore provides proof of concept that up-regulation of NPQ can increase WUE, and as such is an important step towards future engineering of crops with improved performance under drought.

## Teaser

Overexpression of *PsbS* in tobacco reduces stomatal conductance and increases intrinsic water use efficiency during drought stress without significantly affecting plant size under either drought or control conditions.

## Introduction

In recent years the world has experienced extreme droughts (Gessner *et al.*, 2022; Toreti *et al.*, 2023; Zaveri *et al*., 2023), and drought is recognized as the single greatest reason for loss in agricultural productivity (FAO, 2021). Future climate scenarios predict higher air temperatures that will increase leaf transpiration due to an increase in water-vapor pressure deficit from leaf to air (VPD, a measure of atmospheric drought; Lobell *et al.*, 2014; Ort and Long, 2014). One of the potential solutions for assuring food security in the future is the irrigation of more agricultural lands; however, this is not sustainable since agriculture already accounts for the use of 90% of total global freshwater and there are already regions that are suffering from water depletion and restrictions (Scanlon *et al.*, 2012; Dalin *et al.*, 2017). Water supply limitations are therefore likely to impose increasing restrictions on future crop production, underlining a need for crops that require less water per mass of yield.

While stomatal pores typically occupy only up to a few percent of the leaf surface area, they are responsible for 95% of all gas exchange between the leaf and the atmosphere (Kang *et al.*, 2007). Hence, stomatal opening and closing play a crucial role in photosynthesis, transpiration, productivity, and stress tolerance. Multiple mechanisms regulate the opening of stomatal pores to allow for dynamic changes in the environment such as light, humidity, and CO_2_ concentration, and in the metabolomic status of the leaf (Lawson and Matthews, 2020). Light is one of the most dynamic environmental signals (Burgess *et al.*, 2016; Kaiser *et al.*, 2018), and stomata respond in a different way to blue and red light (Roelfsema and Hedrich, 2005; Shimazaki *et al.*, 2007). While the blue-light signaling pathway involves the well-studied activity of the PHOTOTROPIN1 (PHOT1) and PHOT2 blue-light receptors (Kinoshita *et al.*, 2001; Sakamoto and Briggs, 2002), the red-light signaling pathway is largely unknown (Lawson and Matthews, 2020). It is believed that the red-light response is driven by a signal coming from the mesophyll that coordinates stomatal behavior with mesophyll carbon assimilation. The exact carrier of this signal is still to be determined; however, the candidates currently under consideration are gaseous vapor phase ions (Mott and Peak, 2013) and aqueous-based signals (Fujita *et al.*, 2013).

The chloroplastic quinone A (Q_A_) is the primary electron acceptor of photosystem II (PSII), and its oxidized state reflects the balance between excitation energy at PSII and the Calvin–Benson cycle rate. Busch (2014) first suggested that the redox state of Q_A_ is an early signal for light-induced stomatal opening. Later, Glowacka *et al.* (2018) successfully showed that reducing excitation pressure at PSII via increased non-photochemical quenching (NPQ) can serve as a direct way to reduce the stomatal opening in response to light by keeping Q_A_ more oxidized. Modification of NPQ was achieved in *Nicotiana tabacum* by constitutive overexpression of *N. benthamiana PHOTOSYSTEM II SUBUNIT S* (*PsbS*), which is known to play a crucial role in the NPQ mechanism and expression of which affects the amplitude of NPQ (Li *et al.*, 2002). The tobacco plants with higher *PsbS* expression showed a reduction in stomatal opening in response to light that resulted in a 25% reduction in intrinsic water use efficiency under well-water conditions in the field.

In this current study, we hypothesized that a mechanism of modulation of the signal for stomatal opening via NPQ will hold on under water-limited conditions. In addition, we examined whether modulation of Q_A_ via NPQ could be a way to reduce whole-plant daily water consumption and how that would affect plant growth and production under drought stress. To do this, we produced *N. tabacum* lines with overexpression of *PsbS* under a strong photosynthesis-related promoter. The strong relationship between *PsbS* and intrinsic water use efficiency that has previously been seen in plants grown under control conditions was also found to hold under water limitation conditions in our study. Under 60% field water capacity we observed a ~11% increase in the number of CO_2_ molecules fixed per molecule of water loss in the transgenic plants. Our results showed that *PsbS*-overexpression reduced water consumption with no consistent effect on growth under drought conditions.

## Materials and methods

### Plant material and recombinant DNA

The coding sequence of *Arabidopsis thaliana PHOTOSYSTEM II SUBUNIT S* (*PsbS*) (AT1G44575) was cloned between the Arabidopsis *GLYCERALDEHYDE 3-PHOSPHATE DEHYDROGENASE A* promoter (AT3G26650) and the *HEAT SHOCK PROTEIN 18.2* terminator (AT5G59720) in the pCAMBIA2300 vector backbone to create the binary plasmid pNPC2. In addition, neomycin phosphotransferase II from *E. coli* was cloned between the nopaline synthase (NOS) promoter and NOS terminator for resistance to kanamycin *in planta*. *Nicotiana tabacum* cv. ‘Samson’ was transformed using the *Agrobacterium tumefaciens*-mediated protocol of leaf discs (Clemente, 2006). Out of 13 independent T_0_ lines, three showed an increase in NPQ level in the light. These were then used in downstream molecular characterization, which showed a single-copy T-DNA insertion in each line based on a digital droplet PCR (Glowacka *et al.*, 2016). Homozygous T_2_ progeny of these lines were used in the subsequent experiments to produce the data presented in this paper, and are hereafter named as NPC2 lines according to the binary plasmid. As the control in all experiments, the corresponding wild type seeds were used and grown alongside the transformants under the same conditions.

### Plant propagation and experimental design

For all experiments, tobacco seeds were sown and germinated on potting mix containing 40% Canadian peat, 40% coarse vermiculite, 15% masonry sand, and 5% screened topsoil (BM2 Germination and Propagation Mix; Berger, Saint-Modeste, Canada) in a PGC20 reach-in growth chamber (CONVIRON, Winnipeg, MB, Canada). The conditions in the growth chamber were maintained at 25/20 °C day/night temperature, with a 16/8 h photoperiod at 500 μmol m^–2^ s^–1^ and 65% humidity. When they were 7 d old, seedlings were transferred into potting trays of 9 × 4 cells filled with the potting mix, which were placed inside a tray and covered with a clear plastic dome until two true leaves had emerged. During this period the plants were watered as needed. In the case of the greenhouse experiment, the germination and growth in potting trays took place in the greenhouse at 25/19 °C day/night, except for the high-throughput phenotyping experiment where both the day and night temperatures were 2 °C higher. The same soil was used in all experiments, and the weight of dried and fully saturated soil was measured to calculate its field water capacity (FWC).

Seedlings at the two-leaf stage were transplanted to 6 l pots (22.9 cm diameter, 21.6 cm high) filled with the potting mix, except for the high-throughput phenotyping experiment where the pots were 5.5 l (22.3 cm diameter, 21.2 cm high). Slow-release fertilizer (Osmocote Plus 15:9:12 N:P:K; The Scotts Co. LLC, Marysville, OH, USA) was added to each pot at a rate of 3.5 g l^–1^, except for plants grown for high-throughput phenotyping where 150 ppm of liquid fertilizer was used every other day (Peter’s 20-10-20 general purpose fertilizer, 25#; Peters Inc., Allentown, PA, USA). The plants were returned to the same growth chamber or greenhouse where the germination took place and allowed to grow for another 2 weeks. Plants were repositioned randomly twice a week and watered as needed to result in ~85% FWC.

When the plants reached the 5-leaf stage, four independent experiments were conducted to examine physiological traits, molecular changes, and growth under water limitation. Drought was induced by limiting the watering to result in (1) 60% FWC for a growth chamber experiment for molecular and photosynthetic phenotyping; (2) 60% FWC for a growth chamber experiment for water consumption phenotyping; (3) 80% FWC or 65% FWC for a greenhouse experiment for high-throughput phenotyping; and (4) 80% FWC, 65% FWC, and no water for a greenhouse experiment for water consumption or growth phenotyping. Tobacco has a low tolerance to drought, to which it responds by shortening its already relatively short period of vegetative growth. Hence, the drought treatments of 65% and 60% FWC were moderate and were chosen to avoid overly detrimental effects of drought stress, and also confounding effects on physiological traits caused by the plants transitioning from the vegetative to the generative stage. In the case of the no-water treatment, plants were allowed to grow an additional 10 d in control conditions before the treatment was started. The duration of the drought treatments was from 6–14 d, during which the weights of the pots were recorded daily. In the case of the high-throughput-phenotyping experiment, during the treatment period the plants were placed in different greenhouse compartments than during the initial growth period and the temperature was maintained at 27/23° C day/night temperature, 30% humidity, and LED lights of 1300 μmol m^–2^ s^–1^ were used to supplement the natural daylight from 06.00 h to 20.00 h.

### Quantification of mRNA and proteins

Five leaf discs of total of 2.9 cm^2^ were sampled from the youngest fully expanded leaf at 5 h after the start of the photoperiod in 13 d of treatment. Proteins and mRNA were extracted from the same tissue using a NucleoSpin RNA/Protein kit (Macherey-Nagel). The extracted mRNA was treated with DNAse using a Turbo DNA-*free* Kit (ThermoFisher Scientific) and then cDNA was synthesized using a SuperScript III First-Strand Synthesis System (ThermoFisher Scientific). RT-qPCR was used to quantify of expression of Arabidopsis *PsbS* (*AtPsbS*: primers 5´-TCGTTGGTCGTGTTGCTATG -3´ and 5´-CTCTGCTTCGTAAATCGGTATCC-3´) and the corresponding native gene (*NtPsbS*: 5´-GGCACAGCTGAATCTTGAAAC-3´ and 5´-CAGGGACAGGGTCATCAATAA -3´) in relation to actin (*NtACTIN*: 5´-CCTCACAGAAGCTCCTCTTAATC -3´ and 5´-ACAGCCTGAATGGCGATATAC -3´) and ELONGATION FACTOR-1-α (*NtEF:* 5´-TGAGATGCACCACGAAGCTC3´ and 5´-CCAACATTGTCACCAGGAAGTG-3´). RT-qPCR cycle numbers for each of these genes are presented in Supplementary Table S1.

Total protein content was estimated using a Protein Quantification Assay kit (Macherey-Nagel, no. 740967.250). Sample extracts containing 1 μg of total protein were separated by electrophoresis using a Mini Protean TGX stain-free gel (Bio-Rad), and blotted to a Millipore Immobilon-P PVDF Membrane (Merck) using semi-dry blotting (Trans-Blot SD, Bio-Rad). Proteins were then immunolabeled using primary antibodies raised against AtPsbS (1:2000 dilution; Agrisera, AS09533) and the 33 kDa protein of the OXYGEN-EVOLVING COMPLEX OF PSII (AtPsbO; 1:20000 dilution; Agrisera, AS06 142-33), followed by incubation of secondary antibodies (1:2500 dilution; Promega, W401B). A protein ladder (Precision Plus Protein Kaleidoscope, Bio-Rad) was used as a size indicator on each gel. Chemiluminescence was imaged using a LI-COR Odyssey scanner. Protein bands were quantified by densitometry using the LI-COR ImageStudio software (ver. 5.2; Supplementary Table S2).

### NPQ kinetics

Leaf discs of 0.32 cm^2^ were sampled from the youngest fully expanded leaf of plants before the start of a drought treatment (35 d after sowing) and again 13 d later (48 d after sowing). The discs were placed in a 96-Well Plate (781611, BrandTech Scientific, USA) with the abaxial side facing up. Moist sponges were added in each well to prevent the samples from drying and to secure the discs in position. After overnight incubation at room temperature, minimum (*F*_o_) and maximum (*F*_m_) fluorescence were imaged using a Closed FluorCam FC 800-C chlorophyll fluorescence imager (Photon Systems Instruments, Drasov, Czech Republic) followed by 10 min illumination with 2000 μmol m^–2^ s^–1^ light and 10 min dark incubation. Saturated pulses (3200 μmol m^–2^ s^–1^ of cool white 6500 K light) with a duration of 800 ms were used to capture maximum fluorescence under light conditions (*F*_m_´). While 3200 μmol m^–2^ s^–1^ was the highest pulse intensity achievable by the fluorescence imager, it still might not have been fully saturating for the tobacco plants used in this study. To overcome this potential issue, the NPQ values of transgenic lines are discussed in relative terms compared to the wild type. The intervals between saturated pulses were as follows (in s): 15, 30, 30, 60, 60, 60, 60, 60, 60, 60, 60, 60, 60, 9, 15, 30, 60,180, and 300. NPQ was calculated according to Bilger and Bjorkman (1994) as:


$$\begin{array}{l} NPQ=\left(F_{m}/F_{m^{\prime }}\;\;\;\;\right)-1 \end{array}$$ (1)

### Photosynthetic gas exchange

Gas exchange measurements were performed simultaneously with chlorophyll fluorescence measurements using a LI-COR LI-6800 system equipped with a 6 cm^2^ leaf chamber and integrated modulated fluorometer. Measurements took place between 7–11 d of drought treatment, and each day an equal number of plants from each genotype was measured in randomized order. All chlorophyll fluorescence measurements were performed using the multiphase flash routine described by Loriaux *et al.* (2013). The light response of leaf net CO_2_ assimilation (*A*_n_), stomatal conductance (*g*_s_), and intercellular CO_2_ concentration (*C*_i_) were measured at the following sequence of light intensities (100% red LEDs, λ-peak at 630 nm): 0, 50, 80, 110, 140, 170, 200, 300, 400, 500, 600, 800, 1000, 1500, and 2000 µmol m^−2^ s ^−1^ incident photon flux density (PFD). Block temperature was controlled at 23°C, [CO_2_] inside the cuvette was maintained at 400 µmol mol^−1^, and leaf-to-air water VPD was controlled to 1.1–1.2 kPa. Leaves were clamped in the leaf cuvette and dark-adapted for 20 min, after which *F*_m_ was measured to allow the calculation of NPQ at the subsequent light intensities. When a steady state was reached, *A*_n_, *g*_s_, and *C*_i_ were logged, and steady-state fluorescence (*F*_s_) and *F*_m_ʹ were measured. The waiting time to log the points was set between 300–600 s. Most of the points were logged in the first 350 s regardless of the genotype. There were three criteria used for stability: fluorescence, *A*_n_, and *g*_s_. The point was logged when the standard deviation over a period of 15 s was less than 1 for fluorescence and for *A*_n_, and the *g*_s_ rate of change was less than 0.05 over the same period. The operating efficiency of whole-chain electron transport (φPSII, or *F*_q_ʹ/*F*_m_ʹ; Genty *et al.*, 1989), and estimation the fraction of ‘open’ PSII centres (with quinone A oxidized; *q*_L_) were calculated as follows:


$$\begin{array}{l} \;\varphi \;PSII=F_{q}\overset{´}{\;}/F_{m}\overset{´}{\;}=\left(F_{m}\overset{´}{\;}F_{s}\right)/F_{m}\overset{´}{\;} \end{array}$$ (2)

$$\begin{array}{l} qL=(1/F_{s}1/F_{m}\overset{´}{\;})/(1/F_{o}\overset{´}{\;}1/F_{m}\overset{´}{\;}) \end{array}$$ (3)

A short far-red pulse to fully oxidize quinone A (Q_A_) was used to obtain a value of minimal fluorescence without dark adaptation (*F*_o_ʹ). An estimate of the Q_A_ redox state was then calculated as 1–*q*_L_. The calculation of this parameter assumes a ‘lake’ model for photosynthetic antenna complexes where antennae are shared between reaction centers (Kramer *et al.*, 2004). The rate of linear electron transport (ETR) was estimated as:


$$\begin{array}{l} ETR=0.843\times \varphi PSII\times PFD\times 0.5 \end{array}$$ (4)

where the leaf absorption for 100% red light and partitioning of the absorbed PFD between the two photosystems are assumed to be 0.843 and 0.5, respectively. The leaf absorbance was assumed to be the same for all the genotypes examined since a previous study on *PsbS*-overexpressing lines of tobacco showed no significant differences between them and the wild type for this parameter (Kromdijk *et al.*, 2016).

To assess the response of *A*_n_ to [CO_2_], leaves were allowed to reach a steady state at a light intensity of 2000 µmol m^−2^ s^−1^ PFD (100% red), with the block temperature controlled at 25°C and [CO_2_] in the airstream set to 400 µmol mol^−1^. [CO_2_] was then successively decreased to 300, 200, 100, and 75 µmol mol^−1^, returned to 400 µmol mol^−1^, and then increased successively to 500, 600, 700, 800, 1000, 1200, and 1500 µmol mol^−1^, and gas exchange and fluorescence parameters were logged when a steady state was attained. Two steady-state readings were taken when [CO_2_] was returned to 400 µmol mol^−1^ to ensure the recovery of Rubisco activity after the measurements at low [CO_2_] and before progression to the high concentrations. The biochemical model of Farquhar *et al.* (1980) was corrected for the temperature at which the measurements were conducted (Sharkey *et al.*, 2007) and *A*_n_/*C*_i_ curves were fitted to determine the maximal carboxylation rate (*V*_cmax_) and the rate of triose phosphate utilization (*TPU*).

### Degree of Rubisco activation

Rubisco extraction and activity assays were carried out according to Fontaine *et al.* (1999). Discs totalling an area of 4.7 cm^2^ were sampled from the youngest fully expanded leaf at 15.00 h after 13 d of drought stress. The discs were ground in a mortar with liquid nitrogen and 75 mg of polyvinylpolypyrrolidone (PVPP, Sigma). The resulting powder was mixed with 1.75 ml of extraction buffer containing 100 mM HEPES-KOH pH 7.5, 2 mM DTT, 5mM EDTA, 1 mM phenylmethylsulfonyl (PMSF), 1 μM leupeptin (all Sigma), 1 μM pepstatin, 5 mM MgCl_2_, 20 μM of 4 amidinophenylmethylsulfonyl fluoride (4-APMSF; all ThermoFisher Scientific), 10% (w/w) polyvinylpyrrolidone 40, 10% (v/v) glycerol (both Sigma), and 10% (w/w) polyethylene glycol 20 (PEG20, Spectrum Chemicals, New Brunswick, NJ, USA). The mixture was centrifuged for 10 min at 15 000 *g* and 4 °C. The supernatant was then desalted using a PD-10 Desalting Column with Sephadex G25 resin (Cytiva, Westborough, MA, USA). The desalted extract was used for Rubisco quantification and activity assays.

Rubisco activity was determined spectrophotometrically by following NADH oxidation at 340 nm at 30 °C in 200 μl of reaction mixture using a Synergy 2 Microplate Reader (Biotek Instruments, Winooski, VT, USA), as described by Fontaine *et al.* (1999). Briefly, the total and initial Rubisco assay mixtures were 100 mM Bicine pH 8, 25 mM NaHCO_3_^–^ (both ThermoFisher Scientific), 0.25 mM NADH (VWR, Radnor, PA, USA), 3.5 mM ATP, 20 mM MgCl_2_, 5 units ml^–1^ glyceraldehyde 3-phosphate dehydrogenase, 5 units ml^–1^ creatine phosphokinase, 5 units ml^–1^ phosphoglycerate kinase, and 5 mM phosphocreatine (all Sigma). Total Rubisco activity was measured after 10 min incubation at 30 °C before starting the reaction, which was initiated by adding 0.5 mM ribulose-1,5-bisphosphate (Sigma). The initial Rubisco activity was measured at 30 °C without 10 min incubation. The results were determined as mM of CO_2_ degraded per second (nanokatal) related to total protein content, and the degree of Rubisco activation was estimated by calculating the ratio of initial to total activity. Total protein content was determined according to Bradford (1976) using BSA (Macherey Nagel) as the standard.

### High-throughput phenotyping

The phenotyping experiment was carried out using a LemnaTec 3D Scanalyzer system (LemnaTec GmbH, Aachen, Germany) at the Nebraska Innovation Campus Phenotyping Facility (University of Nebraska-Lincoln, USA). Four imaging chambers and multiple watering and weighing stations are the core components of the system, integrated with an automatic conveyor-belt system for pot-scale experiments. The imaging chambers include visible, thermal infrared, steady-state fluorescence, and hyperspectral cameras with specially designed illumination systems (Ge *et al.*, 2016). The watering and weighing stations allow for the quantification of changes in pot weight due to water evaporation and/or evapotranspiration, and enable the precise addition of water to a defined weight.

A hyperspectral camera (Headwall Photonics, Fitchburg, MA, USA) is utilized in the hyperspectral imaging chamber. This camera operates at the spectral range from 550–1700 nm, covering parts of the visible spectrum (green to red), the entire near-infrared (NIR) spectrum, and some of the short-wave infrared (SWIR) spectrum. Each spectral image band has an interval of 4.8 nm, resulting in a total of 243 bands for each hyperspectral image cube. During image capture, both the plants and the camera are stationary. The system employs a rotating scanning mirror to sequentially capture each image line, progressing from the top to the bottom of the imaging chamber. Each grayscale image at one wavelength is 320 pixels (horizontal) and 500 pixels (vertical) with a spatial resolution of ~5 × 5 mm.

Tobacco plants were grown for 13 d under either control conditions of 80% FWC or drought conditions of 65% FWC, and hyperspectral image data were collected across the experimental period. Mean spectral reflectance of the entire plant was obtained from the 3D image cubes through data conversion and reflectance calibration (Pandey *et al.*, 2017). Several vegetation indices related to the leaf chlorophyll content and water content were calculated from the mean spectral reflectance, including the water band index [WBI; Van Gaalen *et al.* (2007)], the normalized difference water index [NDWI; Chen *et al.* (2005)], the pigment specific normalized difference [PSNDa; Blackburn (1998)], the chlorophyll index [CI; Gitelson and Merzlyak (1997)], and the widely used normalized difference vegetation indexes [NDVI; Rouse *et al.* (1974)].

### Growth and biomass measurements

At the end of the experiments, plants were harvested for determination of growth and biomass. Height was measured directly and leaf area was determined using a LI-3100C Leaf Area Meter conveyor-belt scanner. Leaves, stems, and root material were each oven-dried at 70 °C to constant weight for determination of dry mass.

### Stomatal traits

Sections of 1 cm^2^ from the adaxial and abaxial surfaces of the youngest fully expanded leaf located between the midrib and the leaf edge were coated with nail polish. After drying for 10 min, each varnish layer was transferred to a glass slide and covered with a coverslip. Images of these leaf prints were obtained using a Nikon Ti-2 Inverted Fluorescence Microscope with a 4× objective lens equipped with a digital camera (Nikon DS-Qi2). The complete 1 cm^2^ area was constructed into a single image using the stitching feature of the Nikon NIS-Element Software set with a 5% overlap. For each biological replicate and each side of the leaf, two images were analysed and considered as technical replicates. The numbers of stomata were counted via the ImageJ software (https://imagej.net/ij/) using the Cell Counter plugin. For each technical replicate, ten 0.5 mm^2^ squares were drawn and stomata that were more than 50% inside each square were counted. Stomatal density was estimated by dividing the number of stomata in each image by the square size. The lengths, widths, and areas of the stomatal complexes were measured from 20 randomly selected structures within the complete 1 cm^2^ leaf image using ImageJ and then the mean was calculated for each technical replicate.

### Statistical analysis

Normality and homoscedasticity were verified using the Shapiro–Wilk and Brown–Forsythe tests, respectively. The dependent variables were square-transformed to meet model assumptions, as required. The effect of genotypes was tested with ANOVA (α=0.05). Data were analysed by means of a linear mixed-effects model with genotypes as fixed factors and biological replicates as random factors (with the plant as a statistical unit). Differences between transgenic lines and the wild type were tested with Dunnett’s post-hoc test. All statistical analyses were performed using R software v. 4.2.1 (www.r-project.org), with the packages lme4 (Bates *et al.*, 2015) for linear mixed-effect models, stats for the Shapiro–Wilk tests, vGWAS (Shen *et al.*, 2012) for homoscedasticity and Dunnett’s tests, and DescTools (https://cran.r-project.org/web/packages/DescTools/).

## Results

### Overexpression of *PsbS* increases NPQ in *Nicotiana tabacum* under drought stress

**Fig. 1.**
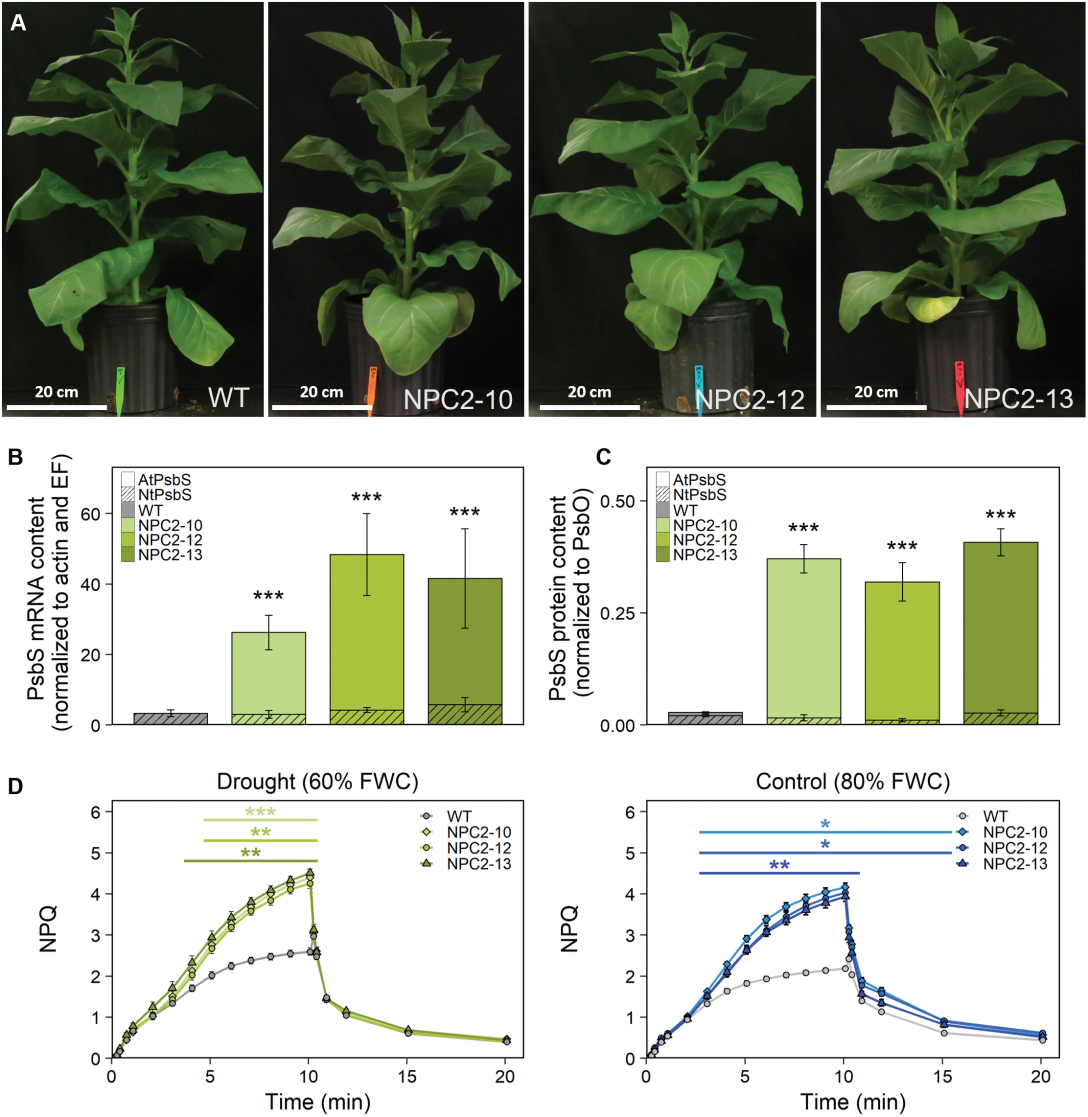
Phenotypes, gene expression, and responses to drought of non-photochemical quenching in wild type *Nicotiana tabacum* and transgenic plants overexpressing *PsbS*. Plants of three independent transgenic lines (NPC2-10, NPC2-12, and NPC2-13) overexpressing Arabidopsis *PsbS* and the corresponding wild-type (WT) were grown under drought conditions of 60% of field water capacity (FWC) and control conditions of 80% FWC. (A) Representative images of plants after 14 d of drought at the end of the experiment. (B) *PsbS* mRNA and (C) PsbS protein contents in fully expanded leaves of plants after 13 d of drought at the end of the experiment. The mRNA content was normalized to the transcript levels of ACTIN and ELONGATION FACTOR (EF) and protein content was normalized to the subunit of the 33 kDa protein of the oxygen-evolving complex of PSII (PsbO). (D) Responses of non-photochemical quenching (NPQ) in the leaves of control and droughted plants to 10 min of light followed by 10 min in the dark. All data are means (±SE) of *n*=6 (B, C) or *n*=10 biological replicates (D). Significant differences compared with the WT were determined using Dunnett’s two-way test: *P≤0.05; **P≤0.01; ***P≤0.001.

Three independent transgenic *N. tabacum PsbS*-overexpressing lines (NPC2-10, NPC2-12, NPC2-13) and the wild type (WT) were grown in a growth chamber at 60% FWC, and *PsbS* mRNA and protein contents were quantified in the leaves after 14 d of the drought treatment (Fig. 1A). Compared to the WT, total *PsbS* transcripts increased by 5, 10, and 8 times in NPC2-10, NPC2-12, and NPC2-13, respectively (Fig. 1B). The differences in mRNA corresponded to 12, 10, and 13 times higher total AtPsbS protein content in NPC2-10, NPC2-12, and NPC2-13, respectively (Fig. 1C). The transgene had a significant effect on NPQ kinetics under drought and control conditions (Fig. 1D). In both treatments, the NPC2 lines showed higher values of NPQ than the WT in the light (+16.5% after 3 min and +90% after 10 min).

### Increased *PsbS* content reduces stomatal conductance and increases intrinsic WUE during drought stress

**Fig. 2.**
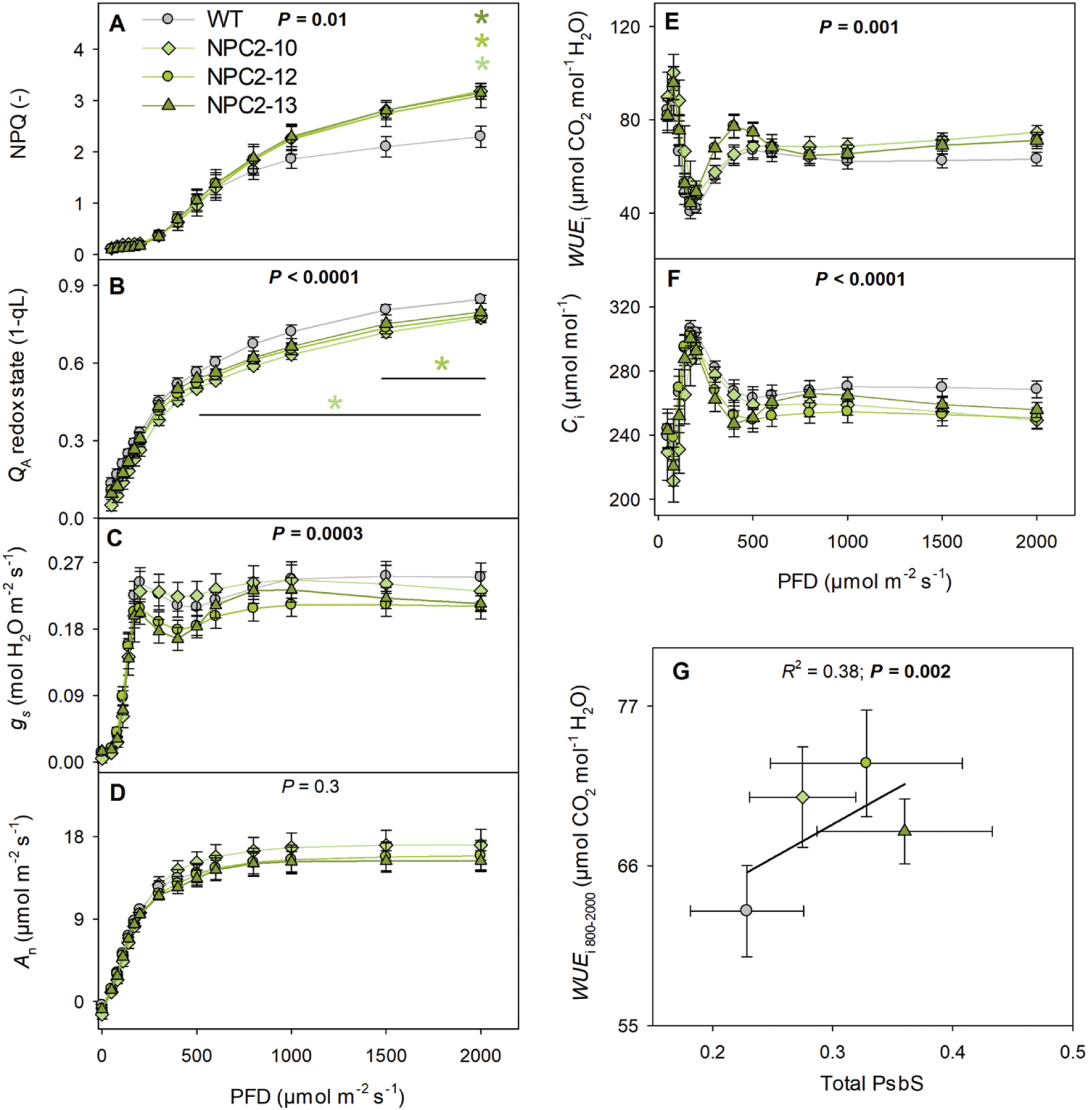
Responses of photosynthesis and intrinsic water use efficiency to drought in wild type *Nicotiana tabacum* and transgenic plants overexpressing *PsbS*. Plants were grown under drought conditions of 60% of field water capacity (FWC). Measurements were taken on fully expanded leaves of three independent transgenic lines (NPC2-10, NPC2-12, and NPC2-13) and the corresponding wild-type (WT) between 7–11 d of the drought treatment. (A) Non-photochemical quenching (NPQ), (B) the redox state of quinone A (Q_A_), (C) stomatal conductance (*g*_s_), (D) net CO_2_ assimilation (*A*_n_), (E) intrinsic water-use efficiency (WUE_i_), and (F) intracellular CO_2_ concentration (*C*_i_) as a function of photon flux density (PFD). (G) Linear correlation between PsbS protein content and WUE_i_. The total PsbS content was normalized to the 33 kDa protein of the oxygen-evolving complex of PSII. WUE_i_ was calculated from the *A*_n_ and *g*_s_ data in the PFD range 800–2000 mol m^–2^ s^–1^. In (A–F), the *P*-values indicate the effect of genotype according to ANOVA, and the asterisks plus lines show significant differences for each transgenic line compared with the WT as determined using Dunnett’s two-way test: *P≤0.05. In (G), *P*-values represent the significance of the linear regression. All data are means (±SE), *n*=7 biological replicates for the WT and *n*=8 biological replicates for the transgenic lines.

During drought stress the higher values of NPQ in the transgenics observed under high light intensities (≥1500 μmol m^–2^ s^–1^; Fig. 2A) corresponded to more oxidized Q_A_ (Fig. 2B). Genotype had a significant effect on the operating efficiency of photosystem II, but not on the electron transport rate (Supplementary Fig. S1). The observed differences in Q_A_ redox state at higher light intensities were reflected in stomatal conductance, which was significantly affected by genotype and lower in the transgenics than the WT (Fig. 2C). Despite the lower *g*_s_ and intracellular CO_2_ concentration in the transgenics (Fig. 2F), the net CO_2_ assimilation (*A*_n_) was not affected by genotype (Fig. 2D). The decrease in *g*_s_ with no changes in *A*_n_ led to an increase in intrinsic water use efficiency (WUE_i_=*A*_n_/*g*_s_) in the transgenics (Fig. 2E). WUE_i_ showed a significantly positive relationship with total PsbS protein content (Fig. 2G). At high light, the *PsbS*-overexpressing lines had on average 9% lower redox state of the Q_A_ pool and 11% higher WUE_i_.

**Fig. 3.**
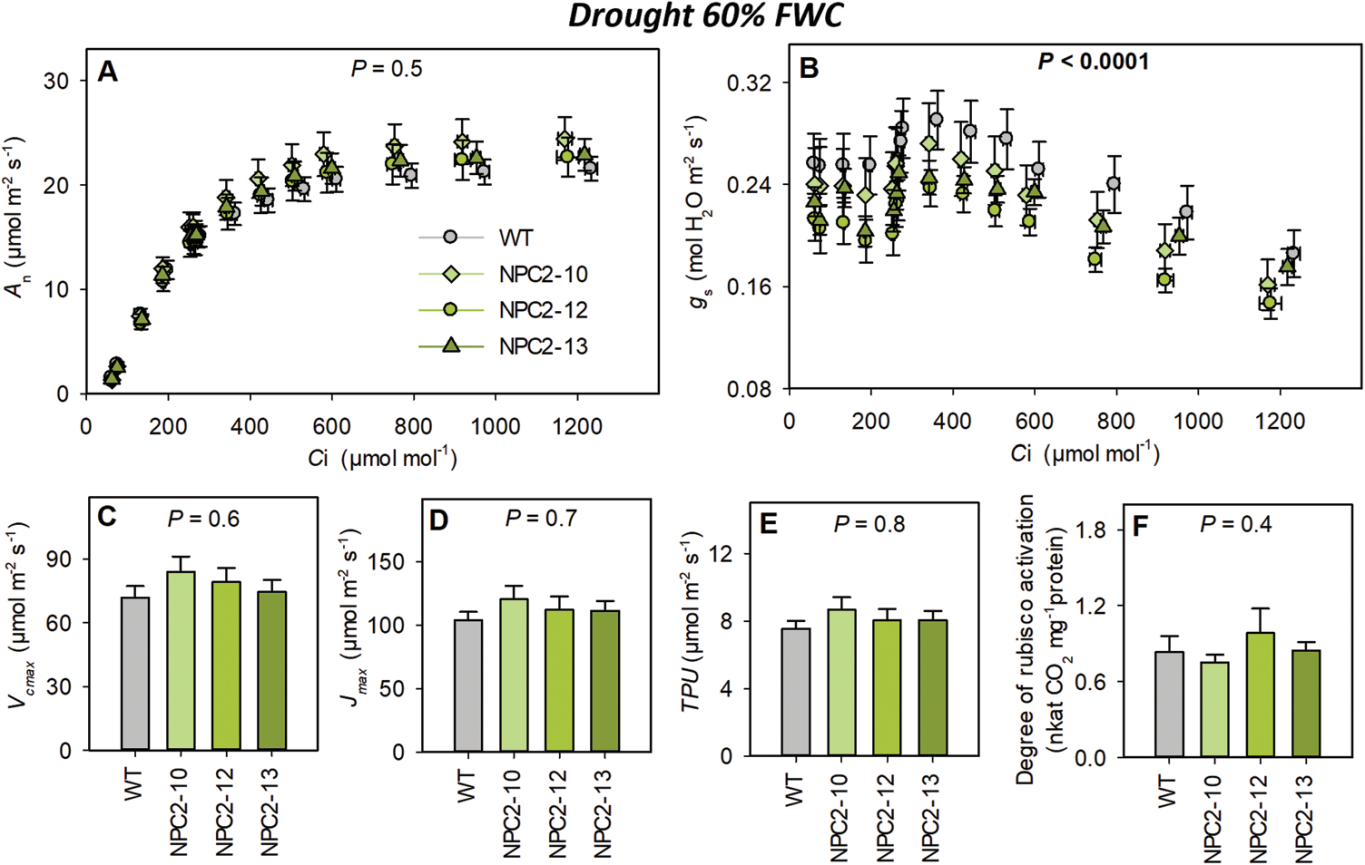
Biochemical capacity of photosynthesis in wild type *Nicotiana tabacum* and transgenic plants overexpressing *PsbS*. Plants were grown under drought conditions of 60% of field water capacity (FWC) for 14 d and measurements were taken on fully expanded leaves of three independent transgenic lines (NPC2-10, NPC-12, and NPC-13) and the corresponding wild-type (WT). (A) Net CO_2_ fixation rate (*A*_n_) and (B) stomatal conductance (*g*_s_) as a function of intercellular CO_2_ concentration (*C*_i_). (C) Maximum ribulose bisphosphate carboxylation capacity (*V*_cmax_) (D) maximum rate of linear electron transport (*J*_max_), (E) rate of triose phosphate utilization (*TPU*), and (F) degree of Rubisco activation. The *P*-values indicate the effect of genotype as determined using ANOVA. All data are means (±SE), *n*=6–8 biological replicates.

The response of *A*_n_ to intracellular CO_2_ (*A*_n_/ *C*_i_; measured at 2000 μmol m^–2^ s^–1^ of light) showed a highly significant effect of genotype on *g*_s_ (Fig. 3B) but not on *A*_n_ (Fig. 3A). Biochemical capacities derived from the *A*_n_/*C*_i_ relationship showed slightly higher (but not significant) values for *V*_cmax_ (Fig. 3C), *J*_max_ (Fig. 3D), and *TPU* in the NPC2 lines than in the WT. The degree of Rubisco activation was not different among the four examined lines (Fig. 3F). There was no consistent effect of increased *PsbS* expression on stomatal anatomy and density (Supplementary Fig. S2). Stomatal density was lower on the adaxial side in all three transgenic lines (by ~10%) with significant reductions in NPC2-12 and NPC2-13. Conversely, on the abaxial side the same two lines showed significant increases in stomatal density when compared to the WT (by 12%). NPC2-13 showed a significant reduction in stomatal width (11%, abaxial side), length (5%, abaxial side), and stomatal area (12%, adaxial side and 13%, adaxial side), but no differences were found in the other two transgenic lines. NPC2-10 and NPC2-12 showed significant increases in the ratio between stomata on the abaxial and adaxial sides of the leaf (20%).

### *PsbS*-overexpressing lines show reduced water requirements under both drought and control conditions

**Fig. 4.**
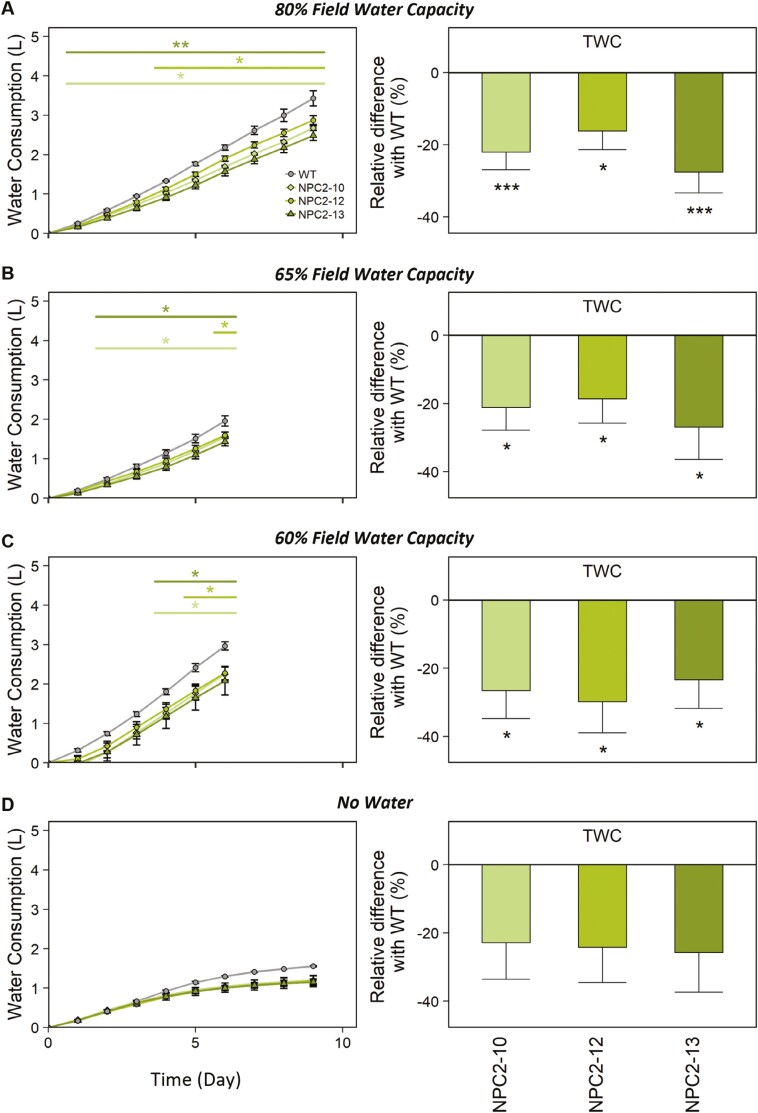
Responses of whole-plant water consumption to different levels of drought in wild type *Nicotiana tabacum* and transgenic plants overexpressing *PsbS*. Plants of three independent transgenic lines (NPC2-10, NPC-12, and NPC-13) and the corresponding wild-type (WT) were grown under conditions of (A) 80% field water capacity (FWC), (B) 65% FWC, (C) 60% FWC, and (D) no water for 9 d (detrimental drought). For each treatment, the water consumption as a function of time is shown on the left, and the relative difference in total water consumption (TWC, %) compared with the WT is shown on the right. The plants in (A, B, D) were grown in a greenhouse whilst those in (C) were in a growth chamber. Significant differences compared with the WT were determined using Dunnett’s two-way test: *P≤0.05; **P≤0.01; ***P≤ 0.001). All data are means (±SE). The number of biological replicates for NPC2-10, NPC2-12, NPC2-13, and WT, respectively, are as follows: (A) 6, 5, 4, and 4; (B) 5, 3, 3, and 4; (C) 5, 4, 4 and 4; (D) 5, 4, 4 and 3.

Significantly positive effects of overexpression of *PsbS* on water saving were observed under the FWC treatments of 80%, 65%, and 60% (Fig. 4; Supplementary Fig. S3). Across these treatments, the NPC2 lines consumed between 10–30% less water than the WT over the duration of the experiments. On average, the daily consumption of the transgenic lines was 62 g (20%), 70 g (25%), and 110 g (26%) less water than that of the WT under 80%, 65%, and 60% FWC, respectively (Fig. 4A–C). In the detrimental drought treatment of no water for 9 d, the cumulative consumption of each of the NPC2 lines was ~ 20% (31 g) less per day than that of the WT, although this was not statistically significant (Fig. 4D).

### *PsbS*-overexpressing lines produce comparable biomasses to the wild type

**Fig. 5.**
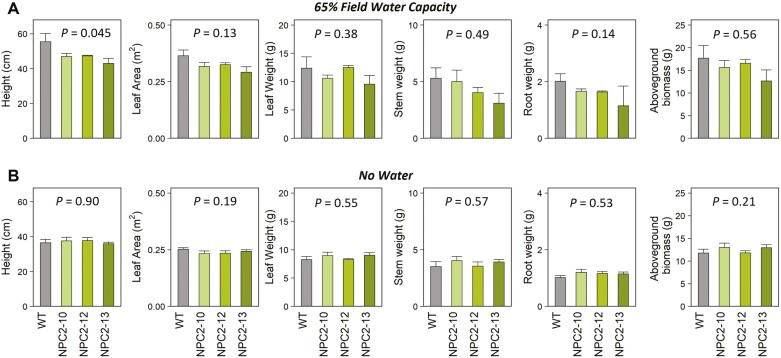
Responses of plant growth parameters to two different drought regimes in wild type *Nicotiana tabacum* and transgenic plants overexpressing *PsbS*. Plants of three transgenic lines (NPC2-10, NPC2-12, NPC2-13) and the corresponding wild type (WT) were grown in a greenhouse under either (A) 65% field water capacity (FWC) or (B) with no water for 9 d (detrimental drought). The *P*-values indicate the effect of genotype as determined using ANOVA; subsequent Dunnett’s two-way tests found no significant differences between the WT and the individual NPC2 lines (*P*>0.05). All data are means (±SE). The number of biological replicates for NPC2-10, NPC2-12, NPC2-13, and WT, respectively, are as follows: (A) 5, 3, 3, and 4; (B) 5, 4, 4, and 3.

When plants were grown under either 65% FWC or 9 d of no water (detrimental drought), there were no consistent effects of *PsbS*-overexpression on plant size (Fig. 5). Under both treatments, stem height, leaf area, leaf weight, stem weight, root weight, and total above-ground biomass were not significantly different between the NPC2 lines and the WT. Whilst ANOVA indicated a significant overall genotype effect on plant height under 65% FWC, Dunnett’s two-way test failed to detect significant differences between the individual transgenic lines and the WT (Fig. 5A). There was no genotype effect on plant height under the detrimental drought treatment (Fig. 5B).

### Physiological and molecular effects of drought at the whole-plant level

**Fig. 6.**
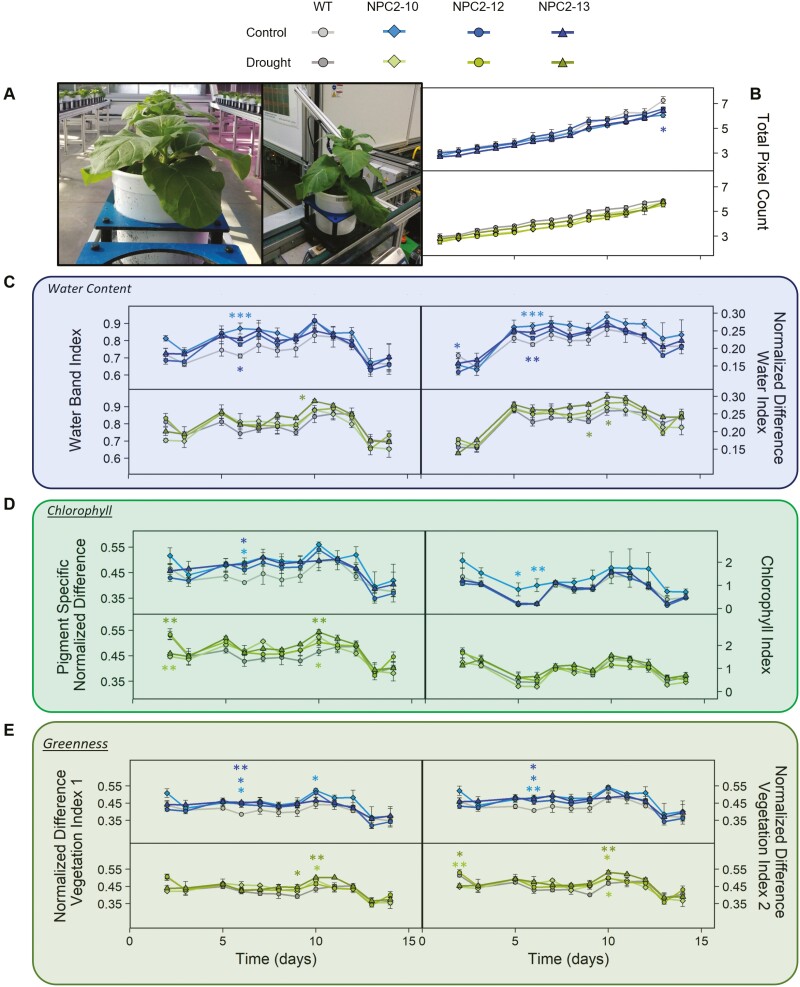
Responses of plant size, water content, chlorophyll content, and greenness to drought in wild type *Nicotiana tabacum* and transgenic plants overexpressing *PsbS*. Plants of three transgenic lines (NPC2-10, NPC2-12, NPC2-13) and the corresponding wild type (WT) were grown in a greenhouse under control conditions of 80% of field water capacity (FWC) or drought (65% FWC) in a high-throughput phenotyping system including hyperspectral imaging. (A) Representative images of plants on the conveyor belts (left) and during automated weighting and watering (right). (B) Daily total pixel counts, providing an indication of plant size. (C–E) Daily variations in different vegetation indexes. (C) Water band index and normalized difference water index, indicating plant water content. (D) Pigment specific normalized difference and chlorophyll index, indicating chlorophyll content. (E) Normalized difference vegetation index 1 and index 2, providing a quantitative means to assess vegetation growth and vigor, including water status, based on reflectance at different wavelengths of near-infrared (NIR) and red light. Index 1 = (NIR_770_–Red_660_)/(NIR_770_+Red_660_), and index 2 = (NIR_800_–Red_670_)/(NIR_800_+Red_670_). All data are means (±SE), *n*=3 biological replicates. Significant differences compared with the WT were determined using Dunnett’s two-way test: *P≤0.05; **P≤0.01; ***P≤0.001.

To monitor physiological changes at the whole-plant level during drought stress, an experiment was conducted in which hyperspectral images were recorded daily to enable the calculation of indexes related to water content, chlorophyll, and total biomass (Fig. 6A). In this experiment, the cumulative water consumption after 13 d in the control (80% FWC) and drought treatments (65% FWC) in the transgenic lines was not significantly lower than that of the WT (Supplementary Fig. S4A). Lower water saving and a general increase in water consumption was observed in this experiment in comparison to our other greenhouse experiments, and this could be due to a lower relative humidity (~40%) that in combination with slightly higher temperature (27 °C) resulted in an increase in VPD (2.4 kPa versus 1.4 kPa).

There were no significant effects of treatment or genotype on plant size at the end of the experiment (Supplementary Fig. S4), and this was in agreement with the plant size estimation based on total pixel counts, which did not differ significantly between the transgenic lines and the WT on any given day during the experiment (Fig. 6B); the only exception was for NPC2-10 in the control treatment, which had a significantly lower pixel count than the WT on the last day. Interestingly, there was a trend of the transgenics having higher water content as estimated from two hyperspectral indexes, namely the water band index and normalized difference water index, and this was particularly apparent between the 5th and 10th day of treatment under control conditions and between the 6th and 10th day under drought conditions (Fig. 6C). The normalized difference water index has been identified as one of the most effective indices for estimating vegetation water content (Chen *et al*., 2005). Some of the differences with the WT became significant on the 6th day under control conditions when water content increased by 23% and 15% in NPC2-10 and NPC2-13, respectively (Supplementary Fig. S5A). Under drought conditions, on the 10th day of treatment the plant water content had significantly increased by 15% in NPC2-13 compared with the WT. Regardless of the treatment, after 10 d the water content started to decline such that all the genotypes reached a similar level by the end of the experiment (Fig. 6C). Chlorophyll content as estimated using the pigment specific normalized difference and the chlorophyll index, and greenness as estimated using two normalized difference vegetation indexes, both showed similar patterns to the water content results (Fig. 6D, E). Normalized difference vegetation indexes provide a quantitative way to assess vegetation growth and vigor, including its water status (Quemada *et al.*, 2021). On the 6th day under control conditions, chlorophyll content had increased by 20% and 17% in NPC2-10 and NPC2-12, respectively Supplementary Fig. S5B), and the greenness index by ~19% across all three transgenic lines (Supplementary Fig. S5C). On the 10th day under drought conditions, chlorophyll content had increased by 12% and 16% in NPC2-10 and NPC2-13, respectively, and the greenness index increased by ~11 % across all three transgenic lines.

## Discussion

For the first time according to our knowledge, this study shows that ectopic expression of *PsbS* makes it possible to reduce stomatal opening under drought conditions with limited effects on CO_2_ assimilation, therefore increasing the intrinsic water use efficiency of the leaf. Constitutive expression of *PsbS* has previously been examined under well-watered control conditions in both the greenhouse and the field, leading to increases in WUE_i_ of ~8% and ~25%, respectively (Glowacka *et al.*, 2018). The strong relationships between Q_A_ redox state and *g*_s_ and between *PsbS* expression and WUE_i_ that were previously seen in plants grown under control conditions were also found under drought conditions in our study (Fig. 2). Under 60% FWC we observed an increase of ~11% in the number of CO_2_ molecules fixed per molecule of water loss in the NPC2 lines. Intriguingly, the WUE_i_ for the WT grown in a growth chamber both under control (Glowacka *et al.*, 2018) and 60% FWC conditions (this study), were identical at 63 μmol CO_2_ mol^–1^ H_2_O, while the *PsbS*-overexpressing lines had higher WUE_i_ under drought conditions.

In agreement with the expectation that the increase in the leaf-level WUE_i_ would lead to a reduction in whole-plant water consumption, significant water savings were observed for the *PsbS*-overexpressing lines under multiple experiments with different levels of drought (Fig. 4; Supplementary Fig. S3). Over the periods of treatment applied, the cumulative use of water was 10–30% less in the *PsbS-*overexpressing lines than in the corresponding WT. On average, the transgenics consumed 62 g (20%), 70 g (25%), and 110 g (26%) less water per day than the WT at 80%, 65%, and 60% FWC, respectively. While the absolute amount of consumed water might be affected by evaporation from the pots and by increases in plant size, the relative difference in water consumption compared to the WT is not affected by these factors, especially since differences in plant size between the genotypes were lacking at the end of the experiment. Compared with the WT, a mean reduction in water consumption of 20% was observed in NPC2 lines after 9 d of detrimental drought conditions (i.e. no water); however, no significant differences were detected. When plants were grown under drier air and higher VPD in the high-throughput phenotyping experiment, which increased water consumption regardless of genotype, the differences compared to the WT became smaller (Supplementary Fig. S4). Interestingly, even under these conditions, we observed a trend of increase in water content in the NPC2 lines between 5th and 10th day of treatment, as estimated by the use of hyperspectral reflectance (Fig. 6C). Higher turgor of the leaves can prevent chlorophyll degradation, which would explain observed higher values of indexes that relate to chlorophyll content and vegetation greenness (Fig. 6D, E). After the 10th day of treatment, all the indexes started to decline, and very similar values were observed for WT and NPC2 lines at the end of the experiment. We speculate that after the 10th day the plants might have become pot-limited, thereby imposing additional stresses that acted to reduce the differences between the genotypes.

The NPC2 lines did not appear to have any obvious growth differences compared to the WT when subjected to water-limited conditions of 65% FWC. The transgenics showed slight reductions in growth relative to the WT; however, the reductions in above-ground biomass were not significant (Fig. 5) while the savings in water were significant (Fig. 4). In the detrimental drought treatment where watering was withheld for 9 d, the *PsbS*-overexpressing lines showed a tendency for greater stem weight and above-ground biomass (Fig. 5), similar to the 80% FWC treatment (Supplementary Fig. S4). It might be expected that plants saving more water per day will grow relatively better if the conditions of water limitation intensify. In agreement with this speculation, we did not see a growth advantage in NPC2 lines under 65% FWC when this moderate level of drought stress was maintained over a period of 6 d; however, we saw a tendency for the NPC2 plants to be bigger under detrimental drought (Fig. 5). In this study, the promoter of *3-PHOSPHATE DEHYDROGENASE A* (*GAPA*) was used to drive the overexpression of *PsbS* in the NPC2 lines. Since *GAPA* expression is regulated by light and is essential for maintaining photosynthetic efficiency due to its role in the Calvin–Benson cycle (Simkin *et al.*, 2023), it would be expected to be a strong promoter. Indeed, a similar increase of ~50% in NPQ level was observed at the last point of the NPQ response to PFD (Fig. 2A; 2000 μmol m^–2^ s^–1^ of light) for the NPC2 lines examined here and in tobacco lines overexpressing *PsbS* under a strong constitutive viral promoter in our previous study (Glowacka *et al.*, 2018). The strong effect of *PsbS*-overexpression on NPQ kinetics (Fig. 1E) could limit the energy available for leaf CO_2_ assimilation under fluctuating light conditions (Hubbart *et al.*, 2012, 2018), which might diminish the growth advantage potentially conferred by our genome modification. In addition, a reduction in stomatal conductance might lead to a reduction in evapotranspiration (Bernacchi *et al.*, 2007).

Water use efficiency is a constraint in the production of resilient and productive crops (Leakey *et al.*, 2019), but improved WUE_i_ often comes at the expense of CO_2_ assimilation (Blum, 2009; Kelly *et al.*, 2012; Takemiya *et al.*, 2013; Lawson and Blatt, 2014; Wang *et al.*, 2019). Here, we have shown that under drought conditions, *PsbS*-overexpressing lines can achieve similar CO_2_ assimilation to the WT despite having lower *g*_s_ that slightly limited the CO_2_ availability via reduced *C*_i_ (Fig. 2). One of the possible mechanisms to compensate for lower CO_2_ concentration inside the leaf is to up-regulate the biochemical capacity of the Calvin–Benson cycle. Indeed, the NPC2 lines showed trends of higher *V*_cmax_, *J*_max_, and *TPU* under drought (Fig. 3). Positive trends between *PsbS* expression and *V*_cmax_, *J*_max_, and *TPU* have also previously been observed under non-drought conditions in rice, tobacco and wheat (Hubbart *et al.*, 2012; Glowacka *et al.*, 2018; Mega *et al.*, 2019).

Our study provides a proof of concept that through overexpression of *PsbS* it is possible to modify stomatal opening under drought conditions to achieve higher water use efficiency and reduce water consumption at the whole-plant level without significant loss of biomass. It has been shown that ‘water-banking’ might have a positive effect on seed yield in wheat and maize under water-limited environments (Cooper *et al.*, 2014; Mega *et al.*, 2019). Therefore, our results provide an important step towards the potential engineering of crops with improved water use efficiency.

## Supplementary data

The following supplementary data are available at *JXB* online.

Fig. S1. Electron transport rate and photosystem II operating efficiency as a function of incident light intensity in the WT and NPC2 lines under 60% FWC.

Fig. S2. Density and dimensions of stomata in the WT and NPC2 lines under 60% FWC.

Fig. S3. Water consumption in the WT and NPC2 lines under 80% FWC followed by 60% FWC.

Fig. S4. Water consumption and growth parameters in the WT and NPC2 lines under 80% FWC or 65% FWC in the high-throughput plant phenotyping experiment.

Fig. S5. Hyperspectral indexes for the WT and NPC2 lines at selected time-points under 80% FWC or 65% FWC.

Table S1. RT-qPCR cycle numbers for *AtPsbS*, *NtPsbS*, *NtACTIN*, and *NtEF.*

Table S2. Densitometry results for the AtPsbS, NtPsbS, and NtPsbO proteins.

erae113_suppl_Supplementary_Tables_S1-S2_Figures_S1-S5

## Data Availability

All data supporting the findings of this study are available within the paper and within its supplementary data published online.
