# Supplementary material for: Up-regulation of non-photochemical quenching improves water use efficiency and reduces whole-plant water consumption under drought in Nicotiana tabacum
Source: J Exp Bot. 2024 Mar 12;75(13):3959–72. doi: 10.1093/jxb/erae113 (PMC11233411; doi:10.1093/jxb/erae113)
Supplement: erae113_suppl_Supplementary_Tables_S1-S2_Figures_S1-S5 [file erae113_suppl_supplementary_tables_s1-s2_figures_s1-s5.pdf]

Up-regulation of non-photochemical quenching improves water use efficiency and reduces whole-plant water consumption under drought

Benjamin Turc, Seema Sahay, Jared Haupt, Talles de Oliveira Santos, Geng Bai, Katarzyna Glowacka

Fig. S1. Electron transport rate and photosystem II operating efficiency as a function of incident light intensity in the WT and NPC2 lines under 60% FWC.

Fig. S2. Density and dimensions of stomata in the WT and NPC2 lines under 60% FWC.

Fig. S3. Water consumption the WT and NPC2 lines under 80% FWC followed by 60% FWC.

Fig. S4. Water consumption and growth parameters the WT and NPC2 lines under 80% FWC or 65% FWC in the high-throughput plant phenotyping experiment.

Fig. S5. Hyperspectral indexes for the WT and NPC2 lines at selected time-points under 80% FWC or 65% FWC.

Table S1. RT-qPCR cycle numbers for *AtPsbS*, *NtPsbS*, *NtACTIN*, and *NtEF*.

Table S2. Densitometry results for the AtPsbS, NtPsbS, and NtPsbO proteins

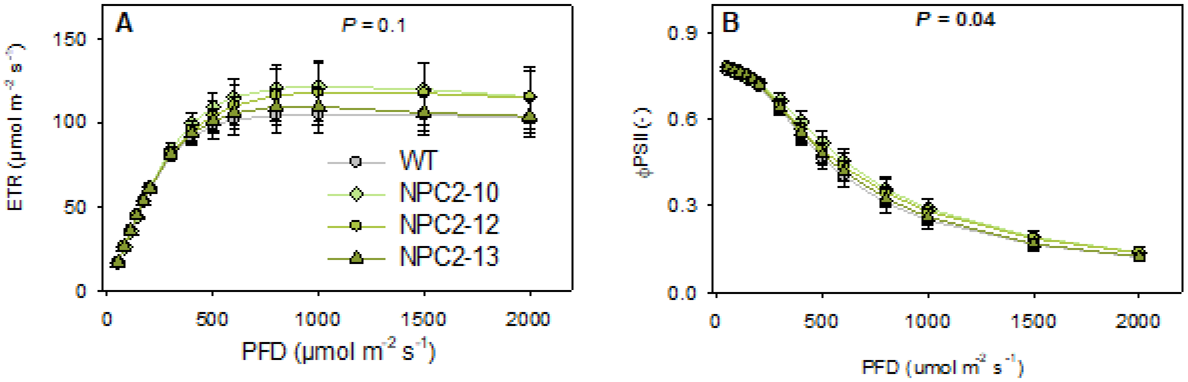

Figure S1 Electron transport rate (ETR) and photosystem II operating efficiency (ΦPSII) as a function of incident light intensity, gas exchange and intrinsic water use efficiency in *Nicotiana tabacum* plants with modified *PsbS* expression grown in drought conditions of 60% of field water capacity. The fully expanded leaves of three independent transgenic lines (NPC2-10, NPC-12 and NPC-13) and corresponding wild-type (WT) were measured. Values represent means ± SE, n = 7 biological replicates for WT and n = 8 biological replicates for transgenic lines. The *P*-values correspond to the effect of the genotype in ANOVA.

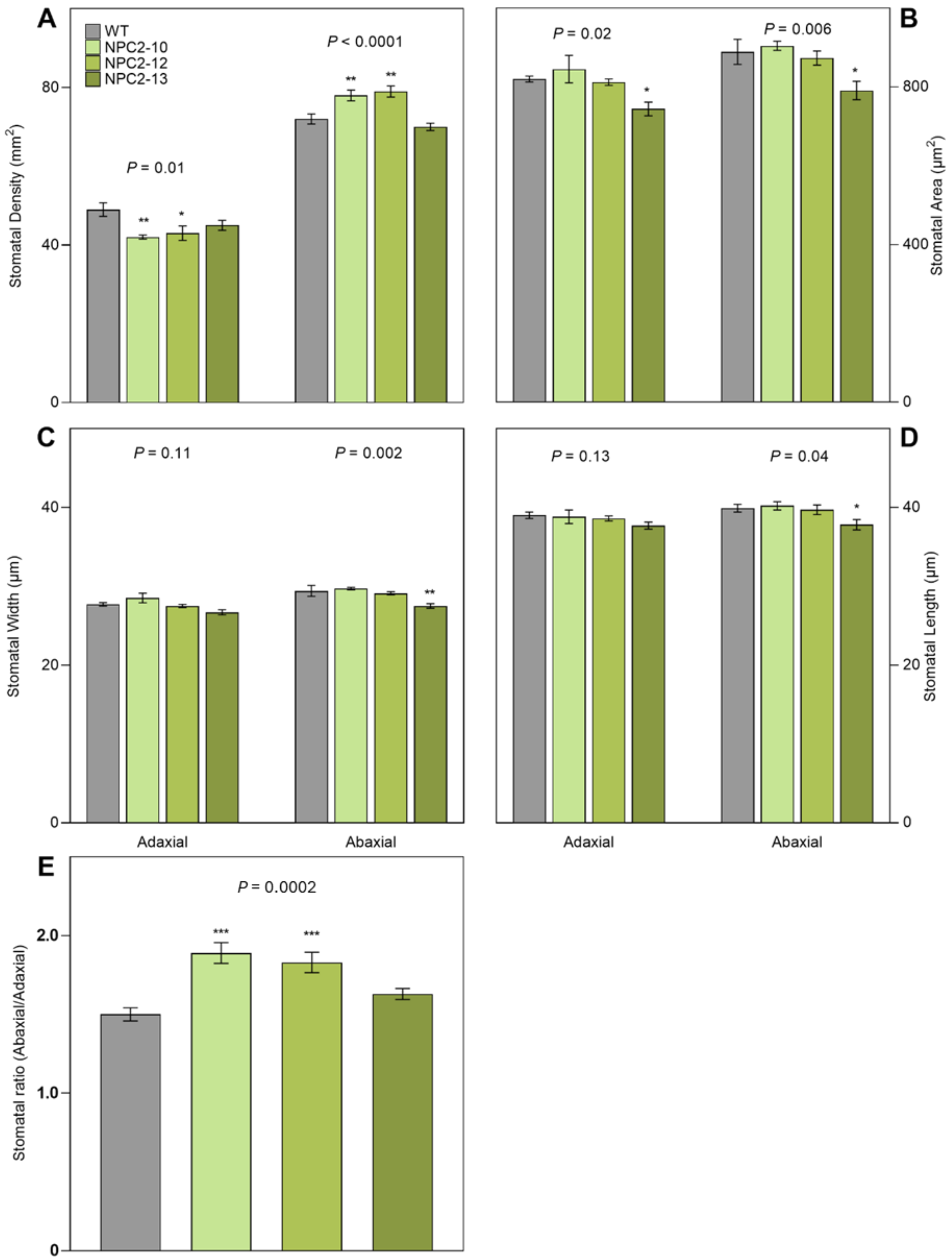

A
WT
NPC2-10
NPC2-12
NPC2-13
P = 0.01
P < 0.0001
Stomatal Density (mm2)
B
P = 0.02
P = 0.006
Stomatal Area (μm2)
C
P = 0.11
P = 0.002
Stomatal Width (μm)
Adaxial
Abaxial
D
P = 0.13
P = 0.04
Stomatal Length (μm)
Adaxial
Abaxial
E
P = 0.0002
Stomatal ratio (Abaxial/Adaxial)

Figure S2. Stomatal density and dimension in *Nicotiana tabacum* plants with modified level of *PsbS* grown in drought conditions of 60% of field water capacity. Three transgenic lines NPC2-10, NPC2-12, NPC2-13 and corresponding wild-type (WT) of tobacco were analyzed. **(A)** Stomatal density, **(B)** stomatal complex area, **(C)** stomatal complex width, **(D)** stomatal complex length and **(E)** ratio between stomata at the abaxial and adaxial side of youngest fully expanded leaves. Error bars show means ± SE (*n* = 6 biological replicates). For stomatal density, each biological replicate is the average of two technical replicates, *i.e.*, leaf prints, with ten 500.000 μm^2 each. For stomatal complex area, width and length, each biological replicate is the average of 20 stomata measured in two leaf prints. P-values indicate the significance of genotype in ANOVA. Asterisks show significant differences from WT (* $P \leq 0.05$; ** $P \leq 0.01$; *** $P \leq 0.01$; Dunnett's two-way test).

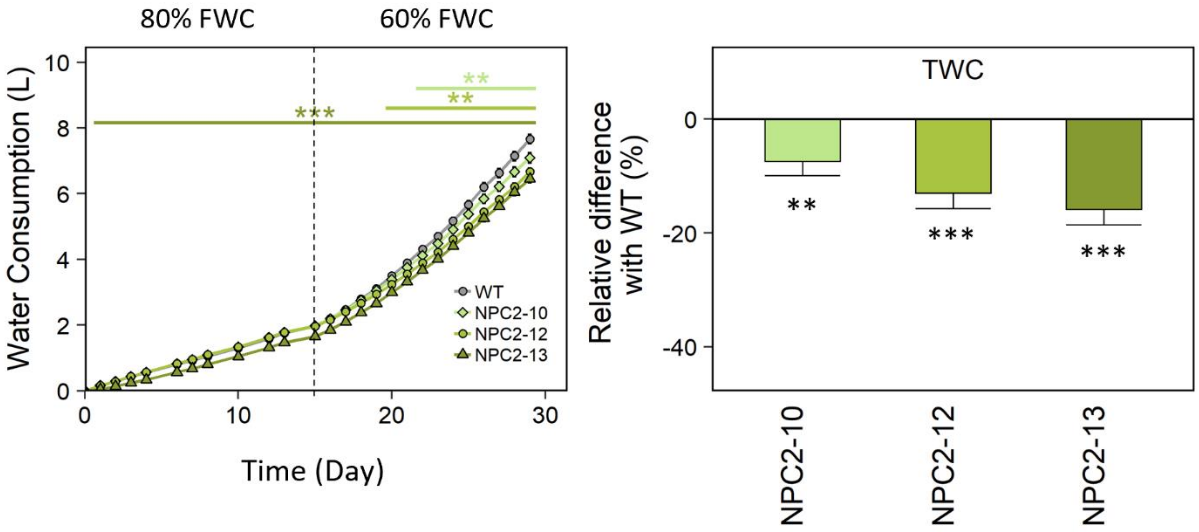

Fig S3. Water consumption of *Nicotiana tabacum* plants with modified *PsbS* expression grown under 80% FWC followed by 60% FWC**.** Three transgenic lines NPC2-10, NPC2-12, NPC2-13 and corresponding wild-type (WT) of tobacco were grown in a growth chamber and subjected to 85% field water capacity (FWC) for 14 days and followed by 14 days with 60% FWC treatment. For each treatment, the water consumption as a function of time (left side panel) and relative difference with WT (%) in total water consumption (TWC; right side panel) are shown. Asterisks/line show significant differences from WT (** $P \leq 0.01$; *** $P \leq 0.001$; Dunnett's two-way test). Values represent means ± SE; n = 10 biological replicates.

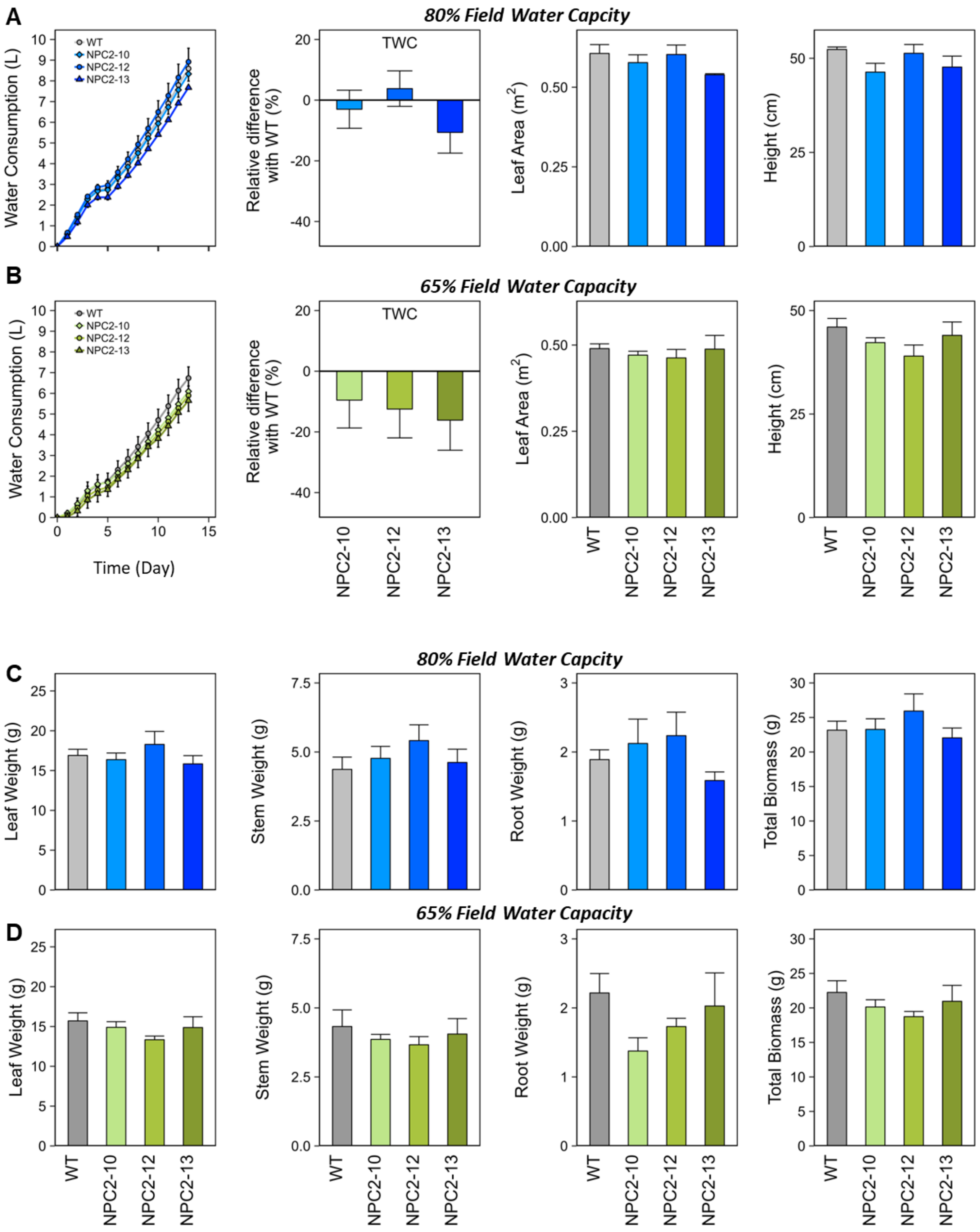

Figure S4. Water consumption and growth of *Nicotiana tabacum* plants with modified *PsbS* expression grown in High-Throughput Plant Phenotyping Greenhouse under two watering regimes. Three transgenic lines NPC2-10, NPC2-12, NPC2-13 and corresponding wild-type (WT) of tobacco were

grown under 80% field water capacity (FWC) and 65% FWC. For each treatment, **(A and B)** the water consumption as a function of time, total water consumption during the treatment (TWC), and **(C and D)** the plant size estimation at the end of the experiment are shown. TWC is expressed as a relative difference with WT (%). The effect of the genotype in ANOVA was not significant ($P > 0.05$). Values represent means ± SE, n = 3 biological replicates.

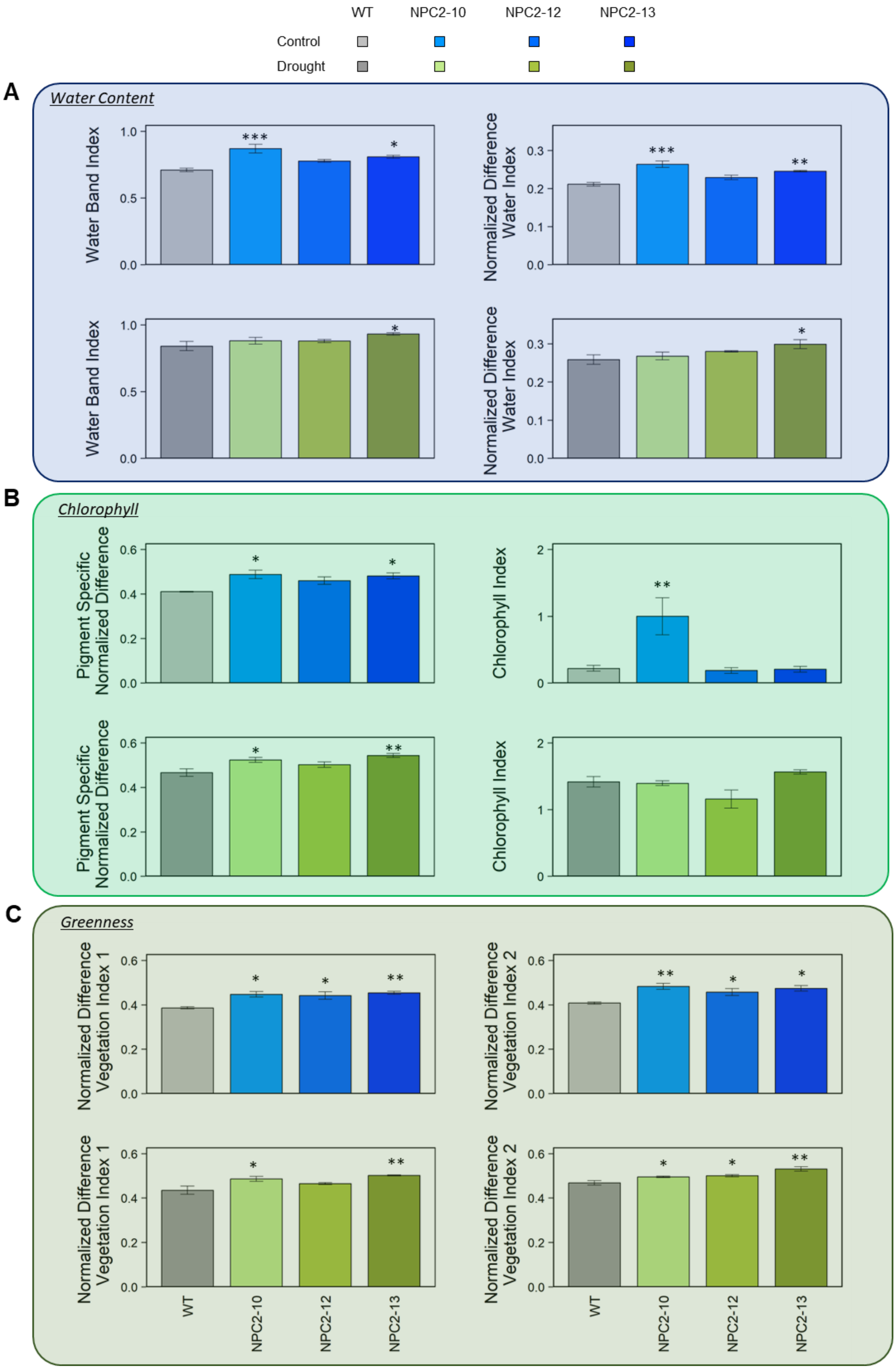
WT
NPC2-10
NPC2-12
NPC2-13
Control
Drought
A
Water Content
Water Band Index
Normalized Difference Water Index
B
Chlorophyll
Pigment Specific Normalized Difference
Chlorophyll Index
C
Greenness
Normalized Difference Vegetation Index 1
Normalized Difference Vegetation Index 2

Figure S5. Hyperspectral indexes related to water and chlorophyll content in leaves and total green biomass for *Nicotiana tabacum* plants with modified *PsbS* expression after 6 days of growth under 80% field water capacity (FWC) and 10 days of 65% FWC conditions when significant differences were first detected**.** Three transgenic lines NPC2-10, NPC2-12, NPC2-13 and corresponding wild-type (WT) of tobacco were analyzed. **(A)** Water Band Index content and Normalized Difference Water Index; **(B)** Pigment Specific Normalized Difference and Chlorophyll Index; **(C)** Normalized Difference Vegetation Index 1 (NDVI1 = (NIR at 770 nm - RED at 660 nm) / (NIR at 770 nm + RED at 660 nm)) and NDVI2 (NDVI2 = NIR at 800 nm - RED at 670 nm) / (NIR at 800 nm + RED at 670 nm)). The changes in hyperspectral indexes during full-time of treatment are presented in Fig. 6. Values represent means ± SE, n = 3 biological replicates. Asterisks show significant differences from WT (* $P \leq 0.05$**; $P \leq 0.01$; *** $P \leq 0.001$; Dunnett's two-way test).

Table S1. RT-qPCR cycle numbers for *AtPsbS*, *NtPsbS*, *NtActin* and *NtEF* (elongation factor α) genes from plants of wild type *N. tabacum* (WT) and three lines expressing *AtPsbS* (NPC2). Samples were taken from greenhouse-grown plants grown under drought conditions. NA – not applicable, signal not detected. Data were used to produce Fig. 1B.

Genotype	Biological replicate	Technical replicate	Cq			
			AtPsbS	*NtPsbS*	*NtActin*	*NtEF*
WT	1	1	NA	27.69	28.66	26.85
WT	1	2	NA	26.95	28.67	26.21
WT	2	1	NA	30.88	29.18	25.67
WT	2	2	NA	30.56	29.18	25.59
WT	3	1	NA	25.81	28.42	25.86
WT	3	2	NA	25.32	27.52	25.86
WT	4	1	NA	24.58	28.23	25.40
WT	4	2	NA	23.80	28.68	24.19
WT	5	1	NA	21.83	25.63	23.10
WT	5	2	NA	21.55	25.02	22.04
WT	6	1	NA	24.44	28.62	25.51
WT	6	2	NA	24.22	28.25	25.30
NPC2-10	1	1	21.35	24.50	28.45	24.66
NPC2-10	1	2	20.86	26.83	29.20	23.83
NPC2-10	2	1	20.37	24.49	27.21	23.00
NPC2-10	2	2	20.46	23.51	27.13	23.04
NPC2-10	3	1	19.57	22.46	27.78	23.07
NPC2-10	3	2	22.38	26.94	26.28	NA
NPC2-10	4	1	23.81	25.09	28.06	22.93
NPC2-10	4	2	19.72	24.40	27.12	23.10
NPC2-10	5	1	23.67	26.05	29.93	26.66
NPC2-10	5	2	23.61	25.40	27.89	24.89
NPC2-10	6	1	23.41	25.51	31.53	27.80
NPC2-10	6	2	23.36	24.87	27.58	26.10
NPC2-12	1	1	19.61	24.38	27.18	23.77
NPC2-12	1	2	20.11	22.81	28.45	24.37
NPC2-12	2	1	19.66	24.08	26.89	24.80
NPC2-12	2	2	19.81	24.72	26.46	25.61
NPC2-12	3	1	20.50	24.61	23.54	30.24
NPC2-12	3	2	20.66	NA	25.71	27.08
NPC2-12	4	1	19.31	21.02	25.35	22.61
NPC2-12	4	2	19.31	23.50	26.15	22.40
NPC2-12	5	1	24.38	27.37	30.63	26.78
NPC2-12	5	2	24.48	26.40	28.40	27.11
NPC2-12	6	1	25.72	27.65	31.71	29.27
NPC2-12	6	2	25.67	25.68	29.17	27.80
NPC2-13	1	1	19.36	22.65	28.20	24.26
NPC2-13	1	2	19.52	22.64	28.68	23.11
NPC2-13	2	1	19.83	22.97	27.52	23.73
NPC2-13	2	2	20.04	22.76	27.02	23.68
NPC2-13	3	1	19.85	21.20	26.19	23.56
NPC2-13	3	2	20.03	21.77	27.21	23.60
NPC2-13	4	1	23.21	23.80	26.50	23.26
NPC2-13	4	2	20.03	24.94	27.42	23.06
NPC2-13	5	1	26.53	28.93	32.31	28.66
NPC2-13	5	2	26.74	28.31	30.79	27.54
NPC2-13	6	1	25.12	28.12	31.75	27.54
NPC2-13	6	2	25.03	28.88	29.30	26.37

Table S2. Densitometry results for AtPsbS, NtPsbS and NtPsbO proteins from leaf protein extract obtained from greenhouse plants grown under drought conditions of wild type *N. tabacum* (WT) and three lines expressing AtPsbS (NPC2). Data were used to produce Fig. 1C.

**Genotypes**	**Biological replicate**	**Densitometry**		
		**AtPsbS**	**NtPsbS**	**NtPsbO**
WT	1	-	0.0016	0.1363
WT	2	-	0.0043	0.1561
WT	3	-	0.0024	0.1253
WT	4	-	0.0065	0.2423
WT	5	-	0.0043	0.2911
WT	6	-	0.0112	0.4226
NPC2-10	1	0.0593	0.0062	0.1452
NPC2-10	2	0.0404	0.0019	0.1103
NPC2-10	3	0.0733	0.0046	0.1666
NPC2-10	4	0.0864	0.0019	0.2979
NPC2-10	5	0.0675	0.0001	0.1738
NPC2-10	6	0.0813	0.0010	0.3475
NPC2-12	1	0.0622	0.0029	0.1704
NPC2-12	2	0.0606	0.0011	0.1509
NPC2-12	3	0.0237	0.0001	0.0646
NPC2-12	4	0.0357	0.0000	0.3217
NPC2-12	5	0.0965	0.0050	0.3402
NPC2-12	6	0.1566	0.0114	0.4859
NPC2-13	1	0.0958	0.0117	0.2006
NPC2-13	2	0.0600	0.0020	0.1599
NPC2-13	3	0.0545	0.0026	0.1435
NPC2-13	4	0.1498	0.0159	0.5405
NPC2-13	5	0.0958	0.0056	0.2937
NPC2-13	6	0.1130	0.0060	0.2523
